# Supplementary material for: Adoption of the 2A Ribosomal Skip Principle to Track Assembled Virions of Pepper Mild Mottle Virus in Nicotiana benthamiana
Source: Plants (Basel). 2024 Mar 22;13(7):928. doi: 10.3390/plants13070928 (PMC11013369; doi:10.3390/plants13070928)
Supplement: Supplementary file 1 [file plants-13-00928-s001.zip › Sequences of viral clones.pdf]

Supplementary Material：

PMMoV：

35S×2

GTAAATTTTTCACAATTTAACAACAACAACACAAACAACAAACAACATTACAAACAAATTAC
AACTACA(PMMoV-5’ UTR)

ATGGCTTACACACAACAAGCTACCAACGCCGCATTAGCAAGTACTCTCCGAGGGAACAACC
CCTTGGTGAACGATCTTGCTAATCGGAGACTGTACGAATCAGCGGTCGAACAATGCAATGC
ACATGACCGCAGGCCCAAGGTTAATTTTTTAAGGTCGATAAGCGAAGAGCAGACGCTTATC
GCAACTAAGGCCTACCCTGAGTTCCAAATTACGTTCTACAACACGCAGAACGCTGTGCACAG
TCTCGCAGGTGGACTTCGGTCTTTGGAACTAGAATACTTGATGATGCAGATCCCCTATGGTT
CAACGACATACGATATCGGGGGAAATTTTGCTGCTCACATGTTTAAAGGTCGTGACTACGTT
CATTGTTGCATGCCTAATATGGACTTACGTGACGTCATGCGTCACAATGCTCAAAAGGATAG
CATTGAACTGTACCTTTCAAAGCTTGCGCAAAAGAAAAAGGTAATACCGCCATATCAAAAG
CCAGTCTTTGATAAATACACGGACGATCCGCAATCGGTAGTGTGCTCGAAACCTTTCCAGCA
CTGCGAAGGCGTTTCGCACTGCACGGATAAAGTATACGCTGTCGCTTTGCACAGTTTATACG
ACATTCCAGCAGATGAATTTGGTGCAGCACTTCTGAGGAGAAATGTTCATGTCTGTTATGCT
GCCTTCCACTTTTCTGAGAATCTTCTTTTAGAAGATTCGTATGTTAGTCTTGACGACATAGGC
GCTTTCTTCTCGAGAGAGGGTGATATGTTGAACTTTTCCTTTGTAGCAGAGAGTACTTTAAAT
TATACTCATTCCTATAGTAATGTGCTTAAGTATGTGTGTAAGACTTACTTCCCCGCTTCTAGT
AGAGAAGTGTACATGAAGGAGTTTTTGGTAACTAGGGTAAATACTTGGTTTTGTAAGTTTTC
AAGGTTAGATACCTTTGTACTATATAGAGGTGTGTACCACAGAGGTGTAGACAAGGAGCAA
TTTTATAGTGCAATGGAAGATGCTTGGCATTACAAAAAGACTTTGGCGATGATGAATAGCGA
AAGGATCCTCTTGGAGGATTCATCGTCTGTTAATTATTGGTTTCCAAAGATGAAAGATATGG
TGATAGTACCTTTGTTCGACGTATCTTTACAGAACGAGGGGAAAAGGTTAGCAAGAAAGGA
GGTTATGGTCAGCAAGGACTTCGTTTATACTGTGCTTAATCATATTCGCACATACCAGTCGA
AAGCGCTTACTTACGCCAATGTATTATCGTTCGTTGAGTCGATAAGATCAAGAGTGATAATT
AATGGGGTGACTGCTCGCTCAGAGTGGGATGTGGACAAGGCTTTGTTGCAGTCTCTGTCAAT
GACCTTTTTCTTGCAGACCAAATTGGCCATGCTCAAAGATGACCTCGTGGTTCAAAAATTTC
AAGTGCATTCTAAATCGCTCACTGAATATGTCTGGGATGAGATTACTGCCGCTTTTCACAACT
GTTTTCCTACAATCAAGGAGAGGTTGATTAACAAGAAACTCATAACTGTTTCGGAAAAGGCT
CTTGAAATTAAAGTACCTGACTTATATGTGACTTTCCACGACAGATTAGTTAAAGAGTACAA
GTCTTCAGTGGAAATGCCGGTACTGGACGTTAAAAAGAGCTTGGAAGAAGCGGAAGTGATG
TACAATGCTTTGTCAGAAATCTCAATTCTTAAGGATAGTGACAAGTTTGATGTTGATGTTTTT
TCCCGAATGTGTAATACTTTAGGCGTAGATCCATTGGTGGCAGCAAAGGTAATGGTAGCCGT
GGTTTCAAATGAGAGTGGTTTGACCTTAACGTTTGAGCGGCCTACCGAAGCAAATGTCGCAC
TTGCATTGCAACCGACAATTGCATCAAAGGAGGAAGGTTCATTAAAGATTGTGTCGTCAGAC
GTAGGTGAGTCCTCAATCAAGGAAGTGGTCCGAAAATCGGAGATTTCTATGCTAGGTCTAA
CAGGCAGCACAGTGTCCGATGAGTTCCAAAGAAGTACAGAAATCGAGTCGTTGCAGCAGTT
CCATATGGTATCCACAGAGACGATTATCCGTAAACAGATGCATGCGATGGTGTATACTGGTC
CGCTAAAAGTTCAACAATGCAAGAACTATTTAGACAGCCTAGTAGCCTCGCTCTCTGCTGCG
GTATCAAACCTGAAGAAGATAATCAAGGACACAGCTGCGATAGATCTCGAGACTAAGGAA

AAATTTGGAGTCTACGACGTGTGCCTTAAGAAATGGTTGGTGAAACCTCAATCGAAAGGAC
ATGCTTGGGGTGTGGTGATGGACTCAGACTATAAGTGCTTTGTAGCGCTTCTCACATACGAT
GGCGAGAACATTGTGTGCGGAGAGACATGGCGTAGAGTCGCAGTGAGCTCCGAATCTTTGG
TGTATTCAGATATGGGGAAGATAAGAGCTATACGCTCAGTGCTTAAAGACGGTGAACCCCA
TATAAGTAGTGCAAAGGTTACACTTGTTGATGGTGTTCCAGGTTGCGGAAGACAAAGGAG
ATTCTTTCGAGGGTCAACTTTGACGAAGATCTGGTTCTAGTACCAGGAAAACAGGCTGCCGA
AATGATAAAAAGAAGAGCAAACAGTTCTGGTTTAATCGTGGCGACCAAGGAGAACGTAAG
GACGGTAGACTCTTTCTTAATGAATTACGGTCGAGGTCCGTGCCAATACAAAAGGCTGTTTC
TGGATGAAGGTCTAATGTTACACCCCGGTTGTGTTAATTTTCTGGTTGGCATGTCTCTATGCT
CCGAGGCTTTTGTTTATGGAGACACCCAGCAGATTCCTTACATCAACAGAGTTGCAACTTTTC
CCTATCCTAAGCATTTGAGTCAACTCGAGGTCGATGCTGTTGAGACTCGCAGAACAACATTG
CGGTGTCCGGCTGATATCACCTTCTTCTTGAATCAGAAGTACGAAGGGCAAGTTATGTGCAC
ATCAAGTGTTACACGCTCGGTGTCACACGAGGTCATCCAAGGTGCAGCAGTAATGAATCCA
GTGTCTAAACCACTTAAAGGGAAGGTGATTACATTCACTCAATCAGACAAGTCATTGCTGCT
CTCAAGGGGTTACGAAGATGTGCATACCGTTCATGAGGTGCAAGGGGAAACGTTTGAAGAC
GTCTCATTAGTGAGATTGACGCCTACACCCGTGGGAATAATTTCAAAGCAGAGTCCGCACCT
GTTGGTCTCGTTGTCTAGGCATACAAGGTCAATCAAATATTACACTGTTGTACTAGATGCAG
TCGTTTCAGTGCTTAGAGATTTGGAGTGTGTGAGTAGTTACCTGTTAGATATGTACAAAGTT
GATGTGTCGACTCAATAG(PMMoV-p126)
CAATTACAGATAGAATCGGTGTACAAAGGTGTTAACCTTTTCGTCGCAGCCCCGAAAACAG
GAGATGTTTCTGACATGCAATATTACTATGACAAGTGTTTGCCGGGAAACAGTACTATACTT
AATGAGTATGATGCTGTAACTATGCAAATACGAGAAAATAATTTGAATGTCAAGGATTGTGT
GTTGGATATGTCGAAGTCGGTGCCTCTTCCGAGAGAATCTGAGACGACATTGAAACCTGTG
ATCAGGACTGCTGCTGAAAAACCTCGAAAACCTGGATTGTTGGAAAACTTGGTCGCGATGA
TCAAAAGAAATTTCAACTCTCCCGAATTAATAGGGGTCGTCGACATCGAAGACACCGCTTCT
CTAGTAGTAGATAAGTTTTTTGATGCATACTTTATTAAAGAAAAGAAAAAACCTAAAAATAT
ACCTCTGCTTTCAAGGGCGAGTTTGGAAAGATGGATAGAAAAGCAAGAAAAGTCGACGATT
GGCCAGTTGGCTGATTTTGACTTTATTGATCTTCCAGCCGTTGATCAATATAGGCACATGATC
AAGCAGCAGCCGAAACAGCGTCTAGATCTTAGTATTCAAACTGAATACCCGGCTTTGCAAA
CTATTGTGTATCATAGCAAGAAAATCAATGCGCTTTTTGGTCCTGTATTTTCAGAATTAACAA
GACAACTGCTAGAGTCAATTGACAGTTCGAGATTCATGTTTTATACAAGGAAAACGCCTACA
CAGATCGAAGAGTTTTTCTCAGATCTGGACTCTAATGTTCCTATGGACATATTAGAGCTGGA
CATTTCCAAGTATGACAAATCACAGAACGAATTTCATTGTGCAGTTGAGTATGAGATTTGGA
AAAGGTTAGGCTTAGACGATTTCTTGGCCGAAGTTTGGAAACACGGGCATCGGAAGACAAC
GTTGAAAGACTACACAGCCGGAATAAAAACGTGTTTGTGGTATCAAAGGAAAAGTGGTGAT
GTCACCACATTCATTGGAAACACGATCATTATTGCTGCATGTCTGTCCTCTATGCTACCGATG
GAGAGATTGATTAAAGGTGCCTTTTGTGGTGATGATAGTATACTATACTTTCCAAAGGGCAC
TGATTTCCCTGATATTCAACAGGGTGCAAATCTTCTCTGGAATTTTGAAGCCAAGTTGTTCAG
GAAGAGATATGGTTACTTTTGCGGTAGGTACATAATCCACCATGACAGAGGTTGTATTGTAT
ATTATGACCCTCTAAAATTGATCTCGAAACTCGGTGCTAAACACATCAAGAATAGAGAACAT
TTAGAGGAATTTAGAACCTCTCTTTGTGATGTTGCTGGGTCGTTGAACAATTGTGCGTACTAT
ACACATTTGGACGACGCTGTCGGTGAGGTTATTAAAACCGCACCTCCTGGTTCGTTTGTTTAT
AGAGCATTAGTTAAGTACTTGTGTGATAAAAGGTTGTTTCAAACATTGTTTCTGGAATAA
(PMMoV-Readthrough domain)

ATGGCGTTAGTAGTCAAGGATGACGTTAAGATTTCTGAGTTCATCAATTTGTCTACCGCTGA
GAAATTCTTACCTGCTGTTATGACTTCGGTCAAGACGGTACGAATTTCGAAAGTTGACAAAG
TGATTGCAATGGAAAACGATTCGTTATCCGATGTAGATTTGCTTAAAGGTGTTAAGCTTGTT
AAAGATGGTTATGTGTGTTTGGCAGGGTTAGTTGTGTCCGGGGAGTGGAACCTACCGGACA
ACTGCAGAGGTGGAGTAAGCGTTTGTTTGGTTGACAAGAGAATGCAAAGAGATGACGAAG
CAACACTTGGATCTTATAGAACCAGTGCGGCTAAGAAACGATTTGCCTTCAAATTGATCCCG
AATTATAGCATTACTACCGCCGATGCTGAGAGAAATGTTTGGCAAGTTTTAGTTAATATTAG
AGGTGTTGCCATGGAAAAGGGTTTCTGTCCTTTATCTTTGGAGTTTGTCTCAGTTTGTATTGT
ACACAAATCCAATATAAAATTAGGCTTGAGAGAGAAAATTACTAGTGTGTCGGAAGGAGGA
CCCGTTGAACTTACAGAAGCAGTTGTTGATGAGTTCATCGAATCAGTTCCAATGGCTGACAG
ATTACGTAAATTTCGCAATCAATCCAAGAAAAGAAGTAATAAGTATGTAGGTAAGAGAAAT
GATAATAAGGGTGTGAATAAGGAAAGGAAGCTGTTTGATAAGGTTAGAATTGGGCAGAACT
CGGAGTCATCGGACGCCGAGTCTTCTTCGTTTTAA (PMMoV-Movement protein)
CT
ATGGCTTACACAGTTTCCAGTGCCAATCAATTAGTGTATTTAGGTTCTGTATGGGCCGATCCA
TTAGAGTTACAAAATCTATGTACTTCGGCGTTAGGTAATCAGTTTCAAACACAGCAGGCTAG
AACTACGGTTCAACAGCAGTTCTCTGATGTGTGGAAGACTATACCGACCGCTACAGTTAGAT
TTCCCGCTACTGGTTTCAAAGTTTTCCGATATAATGCCGTGCTAGATTCTCTAGTGTCGGCAC
TTCTCGGAGCCTTTGATACTAGGAATAGGATAATAGAAGTAGAAAATCCGCAAAATCCTAC
AACTGCCGAGACGCTTGATGCGACGAGGCGGGTAGATGATGCGACGGTGGCCATTAGGGC
CAGTATAAGTAACCTCATGAATGAGTTAGTTCGTGGCACGGGAATGTACAATCAAGCTCTGT
TCGAGAGCGCGAGTGGACTCACCTGGGCTACAACTCCTTAG (PMMoV-Coat protein)
ACATGATGGTGTAAATAAGTTGGACGAACGTTAAACGTCCGTGGCGAGTACGATAACTCGT
AGTGTTTTTCCCTCCACTTAAATCGAAGGGTTGTCGTTAGGATGGAACGCAATTAAATACAT
GTGTGACGTGTATTTGCGAACGACGTAATTATTTTCAGGGGTTCGAATCCCCCCCGAACCGC
GGGTAGCGGCCC (PMMoV-3’ UTR)
HDV RZ-NOS terminator

PMMoV-GFP：

35S×2

GTAAATTTTTCACAATTTAACAACAACAACACAAACAACAAACAACATTACAAACAAATTAC
AACTACA(PMMoV-5’ UTR)

ATGGCTTACACACAACAAGCTACCAACGCCGCATTAGCAAGTACTCTCCGAGGGAACAACC
CCTTGGTGAACGATCTTGCTAATCGGAGACTGTACGAATCAGCGGTCGAACAATGCAATGC
ACATGACCGCAGGCCCAAGGTTAATTTTTTAAGGTCGATAAGCGAAGAGCAGACGCTTATC
GCAACTAAGGCCTACCCTGAGTTCCAAATTACGTTCTACAACACGCAGAACGCTGTGCACAG
TCTCGCAGGTGGACTTCGGTCTTTGGAACTAGAATACTTGATGATGCAGATCCCCTATGGTT
CAACGACATACGATATCGGGGGAAATTTTGCTGCTCACATGTTTAAAGGTCGTGACTACGTT
CATTGTTGCATGCCTAATATGGACTTACGTGACGTCATGCGTCACAATGCTCAAAAGGATAG
CATTGAACTGTACCTTTCAAAGCTTGCGCAAAAGAAAAAGGTAATACCGCCATATCAAAAG
CCAGTCTTTGATAAATACACGGACGATCCGCAATCGGTAGTGTGCTCGAAACCTTTCCAGCA
CTGCGAAGGCGTTTCGCACTGCACGGATAAAGTATACGCTGTCGCTTTGCACAGTTTATACG
ACATTCCAGCAGATGAATTTGGTGCAGCACTTCTGAGGAGAAATGTTCATGTCTGTTATGCT
GCCTTCCACTTTTCTGAGAATCTTCTTTTAGAAGATTCGTATGTTAGTCTTGACGACATAGGC
GCTTTCTTCTCGAGAGAGGGTGATATGTTGAACTTTTCCTTTGTAGCAGAGAGTACTTTAAAT
TATACTCATTCCTATAGTAATGTGCTTAAGTATGTGTGTAAGACTTACTTCCCCGCTTCTAGT
AGAGAAGTGTACATGAAGGAGTTTTTGGTAACTAGGGTAAATACTTGGTTTTGTAAGTTTTC
AAGGTTAGATACCTTTGTACTATATAGAGGTGTGTACCACAGAGGTGTAGACAAGGAGCAA
TTTTATAGTGCAATGGAAGATGCTTGGCATTACAAAAAGACTTTGGCGATGATGAATAGCGA
AAGGATCCTCTTGGAGGATTCATCGTCTGTTAATTATTGGTTTCCAAAGATGAAAGATATGG
TGATAGTACCTTTGTTCGACGTATCTTTACAGAACGAGGGGAAAAGGTTAGCAAGAAAGGA
GGTTATGGTCAGCAAGGACTTCGTTTATACTGTGCTTAATCATATTCGCACATACCAGTCGA
AAGCGCTTACTTACGCCAATGTATTATCGTTCGTTGAGTCGATAAGATCAAGAGTGATAATT
AATGGGGTGACTGCTCGCTCAGAGTGGGATGTGGACAAGGCTTTGTTGCAGTCTCTGTCAAT
GACCTTTTTCTTGCAGACCAAATTGGCCATGCTCAAAGATGACCTCGTGGTTCAAAAATTTC
AAGTGCATTCTAAATCGCTCACTGAATATGTCTGGGATGAGATTACTGCCGCTTTTCACAACT
GTTTTCCTACAATCAAGGAGAGGTTGATTAACAAGAAACTCATAACTGTTTCGGAAAAGGCT
CTTGAAATTAAAGTACCTGACTTATATGTGACTTTCCACGACAGATTAGTTAAAGAGTACAA
GTCTTCAGTGGAAATGCCGGTACTGGACGTTAAAAAGAGCTTGGAAGAAGCGGAAGTGATG
TACAATGCTTTGTCAGAAATCTCAATTCTTAAGGATAGTGACAAGTTTGATGTTGATGTTTTT
TCCCGAATGTGTAATACTTTAGGCGTAGATCCATTGGTGGCAGCAAAGGTAATGGTAGCCGT
GGTTTCAAATGAGAGTGGTTTGACCTTAACGTTTGAGCGGCCTACCGAAGCAAATGTCGCAC
TTGCATTGCAACCGACAATTGCATCAAAGGAGGAAGGTTCATTAAAGATTGTGTCGTCAGAC
GTAGGTGAGTCCTCAATCAAGGAAGTGGTCCGAAAATCGGAGATTTCTATGCTAGGTCTAA
CAGGCAGCACAGTGTCCGATGAGTTCCAAAGAAGTACAGAAATCGAGTCGTTGCAGCAGTT
CCATATGGTATCCACAGAGACGATTATCCGTAAACAGATGCATGCGATGGTGTATACTGGTC
CGCTAAAAGTTCAACAATGCAAGAACTATTTAGACAGCCTAGTAGCCTCGCTCTCTGCTGCG
GTATCAAACCTGAAGAAGATAATCAAGGACACAGCTGCGATAGATCTCGAGACTAAGGAA
AAATTTGGAGTCTACGACGTGTGCCTTAAGAAATGGTTGGTGAAACCTCAATCGAAAGGAC
ATGCTTGGGGTGTGGTGATGGACTCAGACTATAAGTGCTTTGTAGCGCTTCTCACATACGAT
GGCGAGAACATTGTGTGCGGAGAGACATGGCGTAGAGTCGCAGTGAGCTCCGAATCTTTGG

TGTATTCAGATATGGGGAAGATAAGAGCTATACGCTCAGTGCTTAAAGACGGTGAACCCCA
TATAAGTAGTGCAAAGGTTACACTTGTTGATGGTGTTCCAGGTTGCGGGAAGACAAAGGAG
ATTCTTTCGAGGGTCAACTTTGACGAAGATCTGGTTCTAGTACCAGGAAAACAGGCTGCCGA
AATGATAAAAAGAAGAGCAAACAGTTCTGGTTTAATCGTGGCGACCAAGGAGAACGTAAG
GACGGTAGACTCTTTCTTAATGAATTACGGTCGAGGTCCGTGCCAATACAAAAGGCTGTTTC
TGGATGAAGGTCTAATGTTACACCCCGGTTGTGTTAATTTTCTGGTTGGCATGTCTCTATGCT
CCGAGGCTTTTGTTTATGGAGACACCCAGCAGATTCCTTACATCAACAGAGTTGCAACTTTTC
CCTATCCTAAGCATTTGAGTCAACTCGAGGTCGATGCTGTTGAGACTCGCAGAACAACATTG
CGGTGTCCGGCTGATATCACCTTCTTCTTGAATCAGAAGTACGAAGGGCAAGTTATGTGCAC
ATCAAGTGTTACACGCTCGGTGTCACACGAGGTCATCCAAGGTGCAGCAGTAATGAATCCA
GTGTCTAAACCACTTAAAGGGAAGGTGATTACATTCACTCAATCAGACAAGTCATTGCTGCT
CTCAAGGGGTTACGAAGATGTGCATACCGTTCATGAGGTGCAAGGGGAAACGTTTGAAGAC
GTCTCATTAGTGAGATTGACGCCTACACCCGTGGGAATAATTTCAAAGCAGAGTCCGCACCT
GTTGGTCTCGTTGTCTAGGCATACAAGGTCAATCAAATATTACACTGTTGTACTAGATGCAG
TCGTTTCAGTGCTTAGAGATTTGGAGTGTGTGAGTAGTTACCTGTTAGATATGTACAAAGTT
GATGTGTCGACTCAATAG(PMMoV-p126)
CAATTACAGATAGAATCGGTGTACAAAGGTGTTAACCTTTTCGTCGCAGCCCCGAAAACAG
GAGATGTTTCTGACATGCAATATTACTATGACAAGTGTTTGCCGGGAAACAGTACTATACTT
AATGAGTATGATGCTGTAACTATGCAAATACGAGAAAATAATTTGAATGTCAAGGATTGTGT
GTTGGATATGTCGAAGTCGGTGCCTCTTCCGAGAGAATCTGAGACGACATTGAAACCTGTG
ATCAGGACTGCTGCTGAAAAACCTCGAAAACCTGGATTGTTGGAAAACTTGGTCGCGATGA
TCAAAAGAAATTTCAACTCTCCCGAATTAATAGGGGTCGTCGACATCGAAGACACCGCTTCT
CTAGTAGTAGATAAGTTTTTTGATGCATACTTTATTAAAGAAAAGAAAAAACCTAAAAATAT
ACCTCTGCTTTCAAGGGCGAGTTTGGAAAGATGGATAGAAAAGCAAGAAAAGTCGACGATT
GGCCAGTTGGCTGATTTTGACTTTATTGATCTTCCAGCCGTTGATCAATATAGGCACATGATC
AAGCAGCAGCCGAAACAGCGTCTAGATCTTAGTATTCAAACTGAATACCCGGCTTTGCAAA
CTATTGTGTATCATAGCAAGAAAATCAATGCGCTTTTTGGTCCTGTATTTTCAGAATTAACAA
GACAACTGCTAGAGTCAATTGACAGTTCGAGATTCATGTTTTATACAAGGAAAACGCCTACA
CAGATCGAAGAGTTTTTCTCAGATCTGGACTCTAATGTTCCTATGGACATATTAGAGCTGGA
CATTTCCAAGTATGACAAATCACAGAACGAATTTCATTGTGCAGTTGAGTATGAGATTTGGA
AAAGGTTAGGCTTAGACGATTTCTTGGCCGAAGTTTGGAAACACGGGCATCGGAAGACAAC
GTTGAAAGACTACACAGCCGGAATAAAAACGTGTTTGTGGTATCAAAGGAAAAGTGGTGAT
GTCACCACATTCATTGGAAACACGATCATTATTGCTGCATGTCTGTCCTCTATGCTACCGATG
GAGAGATTGATTAAAGGTGCCTTTTGTGGTGATGATAGTATACTATACTTTCCAAAGGGCAC
TGATTTCCCTGATATTCAACAGGGTGCAAATCTTCTCTGGAATTTTGAAGCCAAGTTGTTCAG
GAAGAGATATGGTTACTTTTGCGGTAGGTACATAATCCACCATGACAGAGGTTGTATTGTAT
ATTATGACCCTCTAAAATTGATCTCGAAACTCGGTGCTAAACACATCAAGAATAGAGAACAT
TTAGAGGAATTTAGAACCTCTCTTTGTGATGTTGCTGGGTCGTTGAACAATTGTGCGTACTAT
ACACATTTGGACGACGCTGTCGGTGAGGTTATTAAAACCGCACCTCCTGGTTCGTTTGTTTAT
AGAGCATTAGTTAAGTACTTGTGTGATAAAAGGTTGTTTCAAACATTGTTTCTGGAATAA
(PMMoV-Readthrough domain)
ATGGCGTTAGTAGTCAAGGATGACGTTAAGATTTCTGAGTTCATCAATTTGTCTACCGCTGA
GAAATTCTTACCTGCTGTTATGACTTCGGTCAAGACGGTACGAATTTCGAAAGTTGACAAAG
TGATTGCAATGGAAAACGATTCGTTATCCGATGTAGATTTGCTTAAAGGTGTTAAGCTTGTT

AAAGATGGTTATGTGTGTTTGGCAGGGTTAGTTGTGTCCGGGGAGTGGAACCTACCGGACA
ACTGCAGAGGTGGAGTAAGCGTTTGTTTGGTTGACAAGAGAATGCAAAGAGATGACGAAG
CAACACTTGGATCTTATAGAACCAGTGCGGCTAAGAAACGATTTGCCTTCAAATTGATCCCG
AATTATAGCATTACTACCGCCGATGCTGAGAGAAATGTTTGGCAAGTTTTAGTTAATATTAG
AGGTGTTGCCATGGAAAAGGGTTTCTGTCCTTTATCTTTGGAGTTTGTCTCAGTTTGTATTGT
ACACAAATCCAATATAAAATTAGGCTTGAGAGAGAAAATTACTAGTGTGTCGGAAGGAGGA
CCCGTTGAACTTACAGAAGCAGTTGTTGATGAGTTCATCGAATCAGTTCCAATGGCTGACAG
ATTACGTAAATTTCGCAATCAATCCAAGAAAAGAAGTAATAAGTATGTAGGTAAGAGAAAT
GATAATAAGGGTGTGAATAAGGAAAGGAAGCTGTTTGATAAGGTTAGAATTGGGCAGAACT
CGGAGTCATCGGACGCCGAGTCTTCTTCGTTTTAA (PMMoV-Movement protein)

CT

ATGAAGACTAATCTTTTTCTCTTTCTCATCTTTTCACTTCTCCTATCATTATCCTCGGCCGAATT
CAGTAAAGGAGAAGAACTTTTCACTGGAGTTGTCCCAATTCTTGTTGAATTAGATGGTGATG
TTAATGGGCACAAATTTTCTGTCAGTGGAGAGGGTGAAGGTGATGCAACATACGGAAAACT
TACCCTTAAATTTATTTGCACTACTGGAAAACTACCTGTTCCATGGCCAACACTTGTCACTAC
TTTCTCTTATGGTGTTCAATGCTTTTCAAGATACCCAGATCATATGAAGCGGCACGACTTCTT
CAAGAGCGCCATGCCTGAGGGATACGTGCAGGAGAGGACCATCTTCTTCAAGGACGACGG
GAACTACAAGACACGTGCTGAAGTCAAGTTTGAGGGAGACACCCTCGTCAACAGGATCGAG
CTTAAGGGAATCGATTTCAAGGAGGACGGAAACATCCTCGGCCACAAGTTGGAATACAACT
ACAACTCCCACAACGTATACATCATGGCCGACAAGCAAAAGAACGGCATCAAAGCCAACTT
CAAGACCCGCCACAACATCGAAGACGGCGGCGTGCAACTCGCTGATCATTATCAACAAAAT
ACTCCAATTGGCGATGGCCCTGTCCTTTTACCAGACAACCATTACCTGTCCACACAATCTGCC
CTTTCGAAAGATCCCAACGAAAAGAGAGACCACATGGTCCTTCTTGAGTTTGTAACAGCTGC
TGGGATTACACATGGCATGGATGAACTATACAAACATGATGAGCTTTAA (Green fluorescent protein)

CGAGGGGTAGTCAAGATGCATAATAAATAACGGATTGTGTCCGTAATCACACGTGGTGCGT
ACGATAACGCATAGTGTTTTTCCCTCCACTTAAATCGAAGGGTTGTGTCTTGGATCGCGCGG
GTCAAATGTATATGGTTCATATACATCCGCAGGCACGTAATAAAGCGAGGGGTTCG (TMV-3’ UTR)

GGTCGAGGTCGGCTGTGAAACTCGAAAAGGTTCCGGAAAACAAAAAAGAGAGTGGTAGGT
AATAGTGTTAATAATAAGAAAATAAATAATAGTGGTAAGAAAGGTTTGAAAGTTGAGGAAA
TTGAGGATAATGTAAGTGATGACGAGTCTATCGCGTCATCGAGTACGTTTTAA (Segment of TMGMV-subgenome promoter of CP)

CT

ATGGCTTACACAGTTTCCAGTGCCAATCAATTAGTGTATTTAGGTTCTGTATGGGCCGATCCA
TTAGAGTTACAAAATCTATGTACTTCGGCGTTAGGTAATCAGTTTCAAACACAGCAGGCTAG
AACTACGGTTCAACAGCAGTTCTCTGATGTGTGGAAGACTATACCGACCGCTACAGTTAGAT
TTCCCGCTACTGGTTTCAAAGTTTTCCGATATAATGCCGTGCTAGATTCTCTAGTGTCGGCAC
TTCTCGGAGCCTTTGATACTAGGAATAGGATAATAGAAGTAGAAAATCCGCAAAATCCTAC
AACTGCCGAGACGCTTGATGCGACGAGGCGGGTAGATGATGCGACGGTGGCCATTAGGGC
CAGTATAAGTAACCTCATGAATGAGTTAGTTCGTGGCACGGGAATGTACAATCAAGCTCTGT
TCGAGAGCGCGAGTGGACTCACCTGGGCTACAACTCCTTAG (PMMoV-Coat protein)

ACATGATGGTGTAAATAAGTTGGACGAACGTTAAACGTCCGTGGCGAGTACGATAACTCGT
AGTGTTTTTCCCTCCACTTAAATCGAAGGGTTGTCGTTAGGATGGAACGCAATTAAATACAT

GTGTGACGTGTATTTGCGAACGACGTAATTATTTTCAGGGGTTCGAATCCCCCCCGAACCGC
GGGTAGCGGCCC (PMMoV-3’ UTR)

HDV RZ-NOS terminator

PMMoV-GFP^{2A}：

35S×2

GTAAATTTTTCACAATTTAACAACAACAACACAAACAACAAACAACATTACAAACAAATTAC
AACTACA(PMMoV-5’ UTR)

ATGGCTTACACACAACAAGCTACCAACGCCGCATTAGCAAGTACTCTCCGAGGGAACAACC
CCTTGGTGAACGATCTTGCTAATCGGAGACTGTACGAATCAGCGGTCGAACAATGCAATGC
ACATGACCGCAGGCCCAAGGTTAATTTTTTAAGGTCGATAAGCGAAGAGCAGACGCTTATC
GCAACTAAGGCCTACCCTGAGTTCCAAATTACGTTCTACAACACGCAGAACGCTGTGCACAG
TCTCGCAGGTGGACTTCGGTCTTTGGAACTAGAATACTTGATGATGCAGATCCCCTATGGTT
CAACGACATACGATATCGGGGGAAATTTTGCTGCTCACATGTTTAAAGGTCGTGACTACGTT
CATTGTTGCATGCCTAATATGGACTTACGTGACGTCATGCGTCACAATGCTCAAAAGGATAG
CATTGAACTGTACCTTTCAAAGCTTGCGCAAAAGAAAAAGGTAATACCGCCATATCAAAAG
CCAGTCTTTGATAAATACACGGACGATCCGCAATCGGTAGTGTGCTCGAAACCTTTCCAGCA
CTGCGAAGGCGTTTCGCACTGCACGGATAAAGTATACGCTGTCGCTTTGCACAGTTTATACG
ACATTCCAGCAGATGAATTTGGTGCAGCACTTCTGAGGAGAAATGTTCATGTCTGTTATGCT
GCCTTCCACTTTTCTGAGAATCTTCTTTTAGAAGATTCGTATGTTAGTCTTGACGACATAGGC
GCTTTCTTCTCGAGAGAGGGTGATATGTTGAACTTTTCCTTTGTAGCAGAGAGTACTTTAAAT
TATACTCATTCCTATAGTAATGTGCTTAAGTATGTGTGTAAGACTTACTTCCCCGCTTCTAGT
AGAGAAGTGTACATGAAGGAGTTTTTGGTAACTAGGGTAAATACTTGGTTTTGTAAGTTTTC
AAGGTTAGATACCTTTGTACTATATAGAGGTGTGTACCACAGAGGTGTAGACAAGGAGCAA
TTTTATAGTGCAATGGAAGATGCTTGGCATTACAAAAAGACTTTGGCGATGATGAATAGCGA
AAGGATCCTCTTGGAGGATTCATCGTCTGTTAATTATTGGTTTCCAAAGATGAAAGATATGG
TGATAGTACCTTTGTTCGACGTATCTTTACAGAACGAGGGGAAAAGGTTAGCAAGAAAGGA
GGTTATGGTCAGCAAGGACTTCGTTTATACTGTGCTTAATCATATTCGCACATACCAGTCGA
AAGCGCTTACTTACGCCAATGTATTATCGTTCGTTGAGTCGATAAGATCAAGAGTGATAATT
AATGGGGTGACTGCTCGCTCAGAGTGGGATGTGGACAAGGCTTTGTTGCAGTCTCTGTCAAT
GACCTTTTTCTTGCAGACCAAATTGGCCATGCTCAAAGATGACCTCGTGGTTCAAAAATTTC
AAGTGCATTCTAAATCGCTCACTGAATATGTCTGGGATGAGATTACTGCCGCTTTTCACAACT
GTTTTCCTACAATCAAGGAGAGGTTGATTAACAAGAAACTCATAACTGTTTCGGAAAAGGCT
CTTGAAATTAAAGTACCTGACTTATATGTGACTTTCCACGACAGATTAGTTAAAGAGTACAA
GTCTTCAGTGGAAATGCCGGTACTGGACGTTAAAAAGAGCTTGGAAGAAGCGGAAGTGATG
TACAATGCTTTGTCAGAAATCTCAATTCTTAAGGATAGTGACAAGTTTGATGTTGATGTTTTT
TCCCGAATGTGTAATACTTTAGGCGTAGATCCATTGGTGGCAGCAAAGGTAATGGTAGCCGT
GGTTTCAAATGAGAGTGGTTTGACCTTAACGTTTGAGCGGCCTACCGAAGCAAATGTCGCAC
TTGCATTGCAACCGACAATTGCATCAAAGGAGGAAGGTTCATTAAAGATTGTGTCGTCAGAC
GTAGGTGAGTCCTCAATCAAGGAAGTGGTCCGAAAATCGGAGATTTCTATGCTAGGTCTAA
CAGGCAGCACAGTGTCCGATGAGTTCCAAAGAAGTACAGAAATCGAGTCGTTGCAGCAGTT
CCATATGGTATCCACAGAGACGATTATCCGTAAACAGATGCATGCGATGGTGTATACTGGTC
CGCTAAAAGTTCAACAATGCAAGAACTATTTAGACAGCCTAGTAGCCTCGCTCTCTGCTGCG
GTATCAAACCTGAAGAAGATAATCAAGGACACAGCTGCGATAGATCTCGAGACTAAGGAA
AAATTTGGAGTCTACGACGTGTGCCTTAAGAAATGGTTGGTGAAACCTCAATCGAAAGGAC
ATGCTTGGGGTGTGGTGATGGACTCAGACTATAAGTGCTTTGTAGCGCTTCTCACATACGAT
GGCGAGAACATTGTGTGCGGAGAGACATGGCGTAGAGTCGCAGTGAGCTCCGAATCTTTGG

TGTATTCAGATATGGGGAAGATAAGAGCTATACGCTCAGTGCTTAAAGACGGTGAACCCCA
TATAAGTAGTGCAAAGGTTACACTTGTTGATGGTGTTCCAGGTTGCGGGAAGACAAAGGAG
ATTCTTTCGAGGGTCAACTTTGACGAAGATCTGGTTCTAGTACCAGGAAAACAGGCTGCCGA
AATGATAAAAAGAAGAGCAAACAGTTCTGGTTTAATCGTGGCGACCAAGGAGAACGTAAG
GACGGTAGACTCTTTCTTAATGAATTACGGTCGAGGTCCGTGCCAATACAAAAGGCTGTTTC
TGGATGAAGGTCTAATGTTACACCCCGGTTGTGTTAATTTTCTGGTTGGCATGTCTCTATGCT
CCGAGGCTTTTGTTTATGGAGACACCCAGCAGATTCCTTACATCAACAGAGTTGCAACTTTTC
CCTATCCTAAGCATTTGAGTCAACTCGAGGTCGATGCTGTTGAGACTCGCAGAACAACATTG
CGGTGTCCGGCTGATATCACCTTCTTCTTGAATCAGAAGTACGAAGGGCAAGTTATGTGCAC
ATCAAGTGTTACACGCTCGGTGTCACACGAGGTCATCCAAGGTGCAGCAGTAATGAATCCA
GTGTCTAAACCACTTAAAGGGAAGGTGATTACATTCACTCAATCAGACAAGTCATTGCTGCT
CTCAAGGGGTTACGAAGATGTGCATACCGTTCATGAGGTGCAAGGGGAAACGTTTGAAGAC
GTCTCATTAGTGAGATTGACGCCTACACCCGTGGGAATAATTTCAAAGCAGAGTCCGCACCT
GTTGGTCTCGTTGTCTAGGCATACAAGGTCAATCAAATATTACACTGTTGTACTAGATGCAG
TCGTTTCAGTGCTTAGAGATTTGGAGTGTGTGAGTAGTTACCTGTTAGATATGTACAAAGTT
GATGTGTCGACTCAATAG(PMMoV-p126)
CAATTACAGATAGAATCGGTGTACAAAGGTGTTAACCTTTTCGTCGCAGCCCCGAAAACAG
GAGATGTTTCTGACATGCAATATTACTATGACAAGTGTTTGCCGGGAAACAGTACTATACTT
AATGAGTATGATGCTGTAACTATGCAAATACGAGAAAATAATTTGAATGTCAAGGATTGTGT
GTTGGATATGTCGAAGTCGGTGCCTCTTCCGAGAGAATCTGAGACGACATTGAAACCTGTG
ATCAGGACTGCTGCTGAAAAACCTCGAAAACCTGGATTGTTGGAAAACTTGGTCGCGATGA
TCAAAAGAAATTTCAACTCTCCCGAATTAATAGGGGTCGTCGACATCGAAGACACCGCTTCT
CTAGTAGTAGATAAGTTTTTTGATGCATACTTTATTAAAGAAAAGAAAAAACCTAAAAATAT
ACCTCTGCTTTCAAGGGCGAGTTTGGAAAGATGGATAGAAAAGCAAGAAAAGTCGACGATT
GGCCAGTTGGCTGATTTTGACTTTATTGATCTTCCAGCCGTTGATCAATATAGGCACATGATC
AAGCAGCAGCCGAAACAGCGTCTAGATCTTAGTATTCAAACTGAATACCCGGCTTTGCAAA
CTATTGTGTATCATAGCAAGAAAATCAATGCGCTTTTTGGTCCTGTATTTTCAGAATTAACAA
GACAACTGCTAGAGTCAATTGACAGTTCGAGATTCATGTTTTATACAAGGAAAACGCCTACA
CAGATCGAAGAGTTTTTCTCAGATCTGGACTCTAATGTTCCTATGGACATATTAGAGCTGGA
CATTTCCAAGTATGACAAATCACAGAACGAATTTCATTGTGCAGTTGAGTATGAGATTTGGA
AAAGGTTAGGCTTAGACGATTTCTTGGCCGAAGTTTGGAAACACGGGCATCGGAAGACAAC
GTTGAAAGACTACACAGCCGGAATAAAAACGTGTTTGTGGTATCAAAGGAAAAGTGGTGAT
GTCACCACATTCATTGGAAACACGATCATTATTGCTGCATGTCTGTCCTCTATGCTACCGATG
GAGAGATTGATTAAAGGTGCCTTTTGTGGTGATGATAGTATACTATACTTTCCAAAGGGCAC
TGATTTCCCTGATATTCAACAGGGTGCAAATCTTCTCTGGAATTTTGAAGCCAAGTTGTTCAG
GAAGAGATATGGTTACTTTTGCGGTAGGTACATAATCCACCATGACAGAGGTTGTATTGTAT
ATTATGACCCTCTAAAATTGATCTCGAAACTCGGTGCTAAACACATCAAGAATAGAGAACAT
TTAGAGGAATTTAGAACCTCTCTTTGTGATGTTGCTGGGTCGTTGAACAATTGTGCGTACTAT
ACACATTTGGACGACGCTGTCGGTGAGGTTATTAAAACCGCACCTCCTGGTTCGTTTGTTTAT
AGAGCATTAGTTAAGTACTTGTGTGATAAAAGGTTGTTTCAAACATTGTTTCTGGAATAA
(PMMoV-Readthrough domain)
ATGGCGTTAGTAGTCAAGGATGACGTTAAGATTTCTGAGTTCATCAATTTGTCTACCGCTGA
GAAATTCTTACCTGCTGTTATGACTTCGGTCAAGACGGTACGAATTTCGAAAGTTGACAAAG
TGATTGCAATGGAAAACGATTCGTTATCCGATGTAGATTTGCTTAAAGGTGTTAAGCTTGTT

AAAGATGGTTATGTGTGTTTGGCAGGGTTAGTTGTGTCCGGGGAGTGGAACCTACCGGACA
ACTGCAGAGGTGGAGTAAGCGTTTGTTTGGTTGACAAGAGAATGCAAAGAGATGACGAAG
CAACACTTGGATCTTATAGAACCAGTGCGGCTAAGAAACGATTTGCCTTCAAATTGATCCCG
AATTATAGCATTACTACCGCCGATGCTGAGAGAAATGTTTGGCAAGTTTTAGTTAATATTAG
AGGTGTTGCCATGGAAAAGGGTTTCTGTCCTTTATCTTTGGAGTTTGTCTCAGTTTGTATTGT
ACACAAATCCAATATAAAATTAGGCTTGAGAGAGAAAATTACTAGTGTGTCGGAAGGAGGA
CCCGTTGAACTTACAGAAGCAGTTGTTGATGAGTTCATCGAATCAGTTCCAATGGCTGACAG
ATTACGTAAATTTCGCAATCAATCCAAGAAAAGAAGTAATAAGTATGTAGGTAAGAGAAAT
GATAATAAGGGTGTGAATAAGGAAAGGAAGCTGTTTGATAAGGTTAGAATTGGGCAGAACT
CGGAGTCATCGGACGCCGAGTCTTCTTCGTTTTAA (PMMoV-Movement protein)

CT

ATGAAGACTAATCTTTTTCTCTTTCTCATCTTTTCACTTCTCCTATCATTATCCTCGGCCGAATT
CAGTAAAGGAGAAGAACTTTTCACTGGAGTTGTCCCAATTCTTGTTGAATTAGATGGTGATG
TTAATGGGCACAAATTTTCTGTCAGTGGAGAGGGTGAAGGTGATGCAACATACGGAAAACT
TACCCTTAAATTTATTTGCACTACTGGAAAACTACCTGTTCCATGGCCAACACTTGTCACTAC
TTTCTCTTATGGTGTTCAATGCTTTTCAAGATACCCAGATCATATGAAGCGGCACGACTTCTT
CAAGAGCGCCATGCCTGAGGGATACGTGCAGGAGAGGACCATCTTCTTCAAGGACGACGG
GAACTACAAGACACGTGCTGAAGTCAAGTTTGAGGGAGACACCCTCGTCAACAGGATCGAG
CTTAAGGGAATCGATTTCAAGGAGGACGGAAACATCCTCGGCCACAAGTTGGAATACAACT
ACAACTCCCACAACGTATACATCATGGCCGACAAGCAAAAGAACGGCATCAAAGCCAACTT
CAAGACCCGCCACAACATCGAAGACGGCGGCGTGCAACTCGCTGATCATTATCAACAAAAT
ACTCCAATTGGCGATGGCCCTGTCCTTTTACCAGACAACCATTACCTGTCCACACAATCTGCC
CTTTCGAAAGATCCCAACGAAAAGAGAGACCACATGGTCCTTCTTGAGTTTGTAACAGCTGC
TGGGATTACACATGGCATGGATGAACTATACAAACATGATGAGCTT (Green fluorescent protein)

CAGCTGTTGAATTTTGACCTTCTTAAGCTTGCGGGAGACGTCGAGTCCAACCCTGGGCCC (2A)

ATGGCTTACACAGTTTCCAGTGCCAATCAATTAGTGTATTTAGGTTCTGTATGGGCCGATCCA
TTAGAGTTACAAAATCTATGTACTTCGGCGTTAGGTAATCAGTTTCAAACACAGCAGGCTAG
AACTACGGTTCAACAGCAGTTCTCTGATGTGTGGAAGACTATACCGACCGCTACAGTTAGAT
TTCCCGCTACTGGTTTCAAAGTTTTCCGATATAATGCCGTGCTAGATTCTCTAGTGTCGGCAC
TTCTCGGAGCCTTTGATACTAGGAATAGGATAATAGAAGTAGAAAATCCGCAAAATCCTAC
AACTGCCGAGACGCTTGATGCGACGAGGCGGGTAGATGATGCGACGGTGGCCATTAGGGC
CAGTATAAGTAACCTCATGAATGAGTTAGTTCGTGGCACGGGAATGTACAATCAAGCTCTGT
TCGAGAGCGCGAGTGGACTCACCTGGGCTACAACTCCTTAG (PMMoV-Coat protein)

ACATGATGGTGTAAATAAGTTGGACGAACGTTAAACGTCCGTGGCGAGTACGATAACTCGT
AGTGTTTTTCCCTCCACTTAAATCGAAGGGTTGTCGTTAGGATGGAACGCAATTAAATACAT
GTGTGACGTGTATTTGCGAACGACGTAATTATTTTCAGGGGTTCGAATCCCCCCGAACCGC
GGGTAGCGGCCC (PMMoV-3’ UTR)

HDV RZ-NOS terminator
