# Supplementary figures and images for: Adoption of the 2A Ribosomal Skip Principle to Track Assembled Virions of Pepper Mild Mottle Virus in Nicotiana benthamiana
Source: Plants (Basel). 2024 Mar 22;13(7):928. doi: 10.3390/plants13070928 (PMC11013369; doi:10.3390/plants13070928)

2 day

4 day

6 day

8 day

PMMoV-GFP<sup>2A</sup>

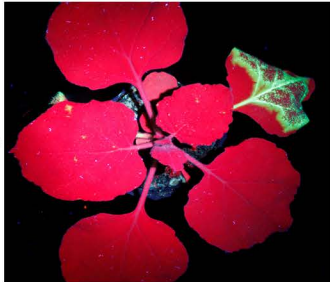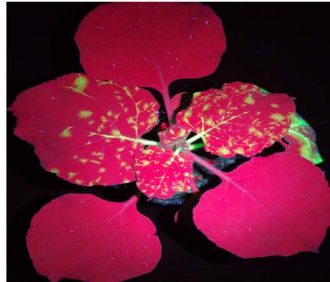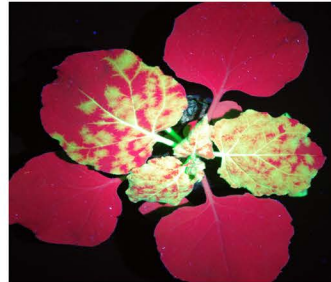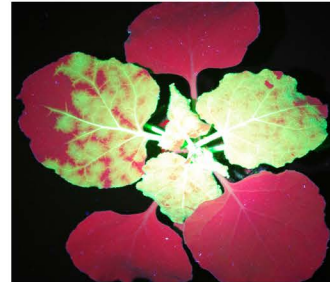

PMMoV-GFP

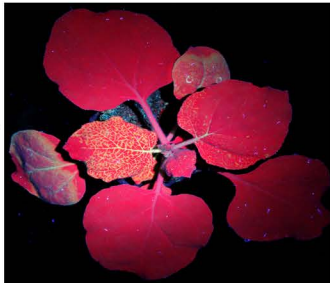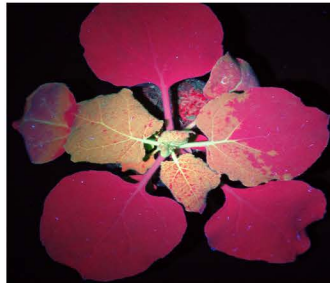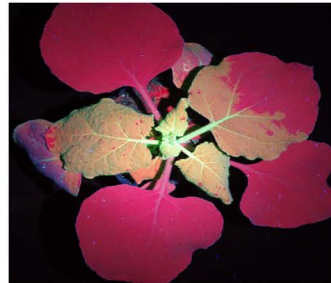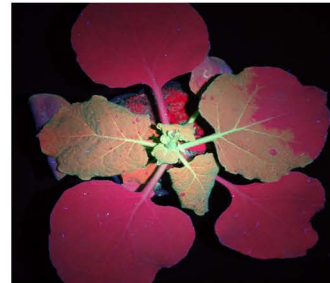

PMMoV

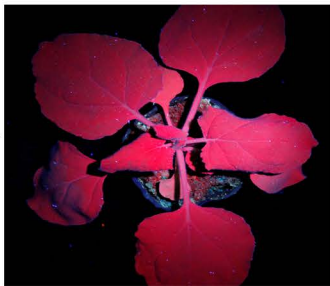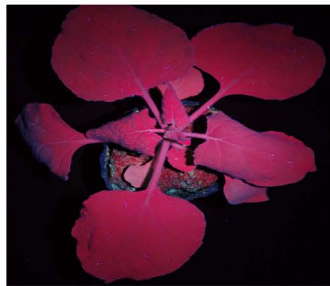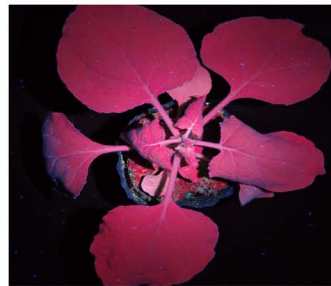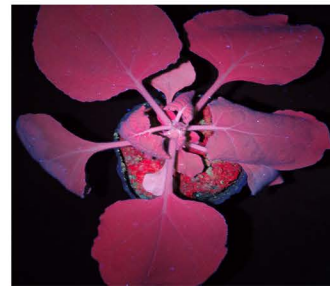

Supplement: Supplementary file 1 [file plants-13-00928-s001.zip › Figure.S1.pdf]
